# Collaboration between orthopaedic surgeons and infection specialists in bone and joint infections

**DOI:** 10.7150/jbji.41662

**Published:** 2019-11-20

**Authors:** Sven Åke Hedström, Lars Lidgren

**Affiliations:** 1 Departments of Infectious Diseases, Lund University, Lund, Sweden; 2 Departments of of Orthopedics, Lund University, Lund, Sweden

## Introduction

In spite of the implementation of certain aseptic procedures in joint prosthetic surgery with so-called clean room air and special body exhaust gowns, as well as the use of multiple antiseptic measures such as preoperative decontamination and antibiotic prophylaxis, orthopaedic infections are still common. Infections caused by work-related or traffic injuries, as well as by blood-borne infections, are demanding, both diagnostically and in terms of treatment. The societal burden is also significant. Data from the Swedish Social Insurance Agency during a 6-year period starting in 1975 reported 1900 orthopaedic infections in Sweden, a country of 8.5 million inhabitants.

For more than 15 years, orthopaedic and infection clinics in Lund, Sweden, have collaborated. Since 1974, this collaboration has become more structured and systematic. A total of 132 patients with orthopaedic complications were treated in the infection clinic in 1980, which corresponds to 14.3% of the infection clinic's capacity, with an average occupation of nine ward beds.

Half of the more difficult orthopaedic cases were admitted to one particular ward, where the staff received additional training on how to care for postoperative patients.

## Types of patients

Three types of patients are currently admitted to the infection clinic.

1. Patients referred by the orthopaedic surgeons from the emergency clinic, or patients directly seeking or being referred to the infection clinic. This group includes patients with, for example, septic arthritis, osteomyelitis, diabetic osteitis, soft tissue infections, and decubital ulcers. These patients have generally not previously been in contact with an orthopaedic surgeon or an infection specialist in the hospital (100 patients).

2. Patients with postoperative infections. These patients have already received primary treatment by an orthopaedic surgeon (14 patients).

3. Referral patients with severe infections. These patients are oftentimes from a wider catchment area or region (18 patients).

## Treatment routines and collaboration

Clinical visits are scheduled at the infection clinic 2 days per week. Usually the orthopaedic surgeon (LL) sees all the patients. Each visit lasts approximately 1-2 hours. The infection specialist (SÅH), who is responsible for orthopaedic patients with infections, participates along with physiotherapists, junior physicians from the ward, and nurses who manage wound care.

By concentrating patients with particularly difficult surgeries mainly into one ward, postoperative care today is on par with that on an orthopaedic ward. Wound treatments such as mechanical rinsing, local treatment with antibiotics, suction irrigation drainage, skin graft dressings, conventional Stryker traction bed treatment, and plaster of Paris and other dressings are just a few examples of what is done at the infection clinic today. These types of wound treatments are time-consuming and often difficult to treat.

In some cases, the permanent in-charge orthopaedic consultants have not performed the surgeries or the interventions of the patients at the infection clinic because of time constraints. This applies mainly to amputations, ulcers with minor infections, and wound revisions. Hand infections are mainly managed by a hand specialist. All of these patients were preoperatively examined by the orthopaedic surgeon who performed the surgery, who also signed off the surgical reports.

The surgeries are performed in a separate room for patients with infections, which is located within the orthopaedic clinic's main operating area. It is crucial that this specific theatre is in the vicinity of the regular surgical theatre, given that it is often necessary to have access to certain surgical instruments and hardware, especially for reoperation of infected implants and fractures.

## Two infection cases after major surgery every week

A retrospective medical record review shows that in 1980, 84 patients with infections as indications had major surgeries, an average of two infection cases after surgery per week. Patients who were identified during surgery to have infections were not included in this review. Forty-eight of the 84 patients had operations performed by the surgeon in charge of the infections. When surgeries have to be redone, for a number of reasons, including psychological, it is deemed crucial that the physician who primarily treated the patient participate in the surgery.

At the infection clinic, information on the possibility of compensation by Swedish patient insurance in the case of unexpected complications, that is, for postoperative infections, is provided by the social worker. This occurs only after discussion with the treating infection specialist or orthopaedic surgeon.

## Competition for rooms

Approximately 75% of all patients diagnosed with orthopaedic infections were admitted to the infection clinic. The rest were treated at the orthopaedic clinic.

In some cases, the infection clinic was fully occupied and patients with postoperative infections could not be immediately transferred. At the orthopaedic clinic, there are three single rooms with the possibility to isolate in each ward, two of these isolation rooms having double doors and ventilation with constant low air pressure.

However, the demand for these rooms is extremely high given that two-thirds of the admitted orthopaedic patients are non-elective cases. Many have, for example, been exposed to trauma or have tumours, thus requiring privacy.

An ideal solution with all infected orthopaedic cases treated at the infection clinic is certainly not possible, and may not even be desirable. However, the current balance in Lund, with care of non-contagious patients at the orthopaedic ward and care of contagious patients at the infection clinic, seems reasonable. The local catchment area for Lund, in addition to the regional responsibility (1.6 million), amounts to 200,000 patients. There are 65 beds at the infection clinic and 109 at the orthopaedic clinic.

## Joint outpatient clinic

Since 1978, a 2-hour joint outpatient clinic has been held every second week at the orthopaedic clinic with infection specialists, orthopaedic surgeons, and, at times, plastic surgeons. Because we believe these routines are valuable, a brief description is given.

Patients are mainly referred by colleagues working at the infection and orthopaedic clinics, but may also be referred from outside. Requests for second opinions and treatment have also been submitted from insurance companies.

Advantages of joint assessments are that treatment plans with input from all specialists involved can be set up, decisions can be made as to the clinic to which the patient should be admitted, and additional assessments/medical follow-ups can be ordered immediately, if applicable.

Furthermore, potential surgical procedures and non-operative treatment are directly discussed with the patient. As an example, in cases with decubital sacral ulcers due to paraplegia, the patient's overall living and social situation can be discussed. Any changes or modifications regarding, for instance, wheelchairs or decubital seat cushions have to be arranged. Before surgery is scheduled, whether the patient is able to stay in a prone position for an extended period must be determined.

In 1980, we had about 80 visits at this outpatient clinic. Fifty-five were new visits and 21 were referral cases. Of the latter cases, eight were set up for operational intervention and the rest given an extensive treatment plan for the respective referral clinic to handle. At 24 of the 80 visits, plastic surgery problems were discussed.

## Educational training courses

This long-term collaboration between orthopaedic and infection clinics in Lund has resulted in a number of valuable new practical treatment principles. We thought it was important to provide information about these experiences and possibly stimulate other units in Sweden or elsewhere to start a similar clinical collaboration. In 1979, we initiated a 3-day training course under the auspices of the Swedish Orthopaedic Society. The participants were accepted only if both the infection and orthopaedic specialists in senior positions from the same hospital applied.

We thereby hoped to initiate an interactive discussion during this course on how collaboration at the specialists' own hospitals could be organized. Furthermore, this service and collaboration would not completely vanish if one of the specialists left and had to be replaced.

Forty-four physicians from 22 central hospital/university clinics attended. In order to see whether the clinics had addressed some of the routines that the course highlighted, we asked the participating clinics the following questions 2 years later, in 1981.

l. Do you routinely transfer patients with bone and joint infections from the orthopaedic ward to the infection clinic?

2. Is there an assigned orthopaedic consultant and infection specialist?

3. Are there joint orthopaedic and infection outpatient visits?

4. Have any of the treatment principles that the course advocated been practiced?

In three of the 22 clinics, no routine transfer of infected patients to the infection clinic had been implemented. At three clinics, no orthopaedic consultants regularly visited the ward despite referral of infected cases.

The joint outpatient visits were implemented at five clinics after the training course; however, 14 of 22 still had no combined outpatient clinic.

Ten clinicians reported that they had received important new information regarding diagnostic modalities and treatment, especially for local treatment and systemic antibiotics. In many cases, the participants pointed out that it was valuable to, for the first time, have the opportunity to discuss in detail the collaboration with a colleague from their own hospital.

## 50 Years later do we need to revisit?

Because of an ageing population, fragility fracture surgeries and arthroplasties have rapidly increased in Sweden while traffic and work-related injuries have decreased. With 40 000 joint replacements and 50 000 fragility fracture surgeries yearly, compounded by approximately 900 infections, this poses a significant societal and humanitarian burden. Emerging bacterial resistance calls for antimicrobial stewardship, and the need for collaboration between infection and orthopaedic specialists is even more important today. Centralization to fewer highly specialized units seems warranted because of the complexity of some cases that need to undergo revision surgery. This could be organized in different ways in specialized wards either at an infection clinic or at an orthopaedic unit. During the first scientific meeting of EBJIS in 1982, a presentation was given by one of the authors (SÅH) on the importance of clinical collaboration. This has, ever since, been a scientific backbone of our society and a necessary foundation in our clinical work in treating bone and joint infections.

